# A stacking-based model for predicting 30-day all-cause hospital readmissions of patients with acute myocardial infarction

**DOI:** 10.1186/s12911-020-01358-w

**Published:** 2020-12-14

**Authors:** Zhen Zhang, Hang Qiu, Weihao Li, Yucheng Chen

**Affiliations:** 1 School of Computer Science and Engineering, University of Electronic Science and Technology of China, No.2006, Xiyuan Ave, West Hi-Tech Zone, 611731 Chengdu, Sichuan PR China; 2 Big Data Research Center, University of Electronic Science and Technology of China, Chengdu, China; 3 Cardiology Division, West China Hospital, Sichuan University, No.17 People’s South Road,Chengdu, 610041 Chengdu, Sichuan PR China; 4 West China Biomedical Big Data Center, West China Hospital, Sichuan University, Chengdu, China

**Keywords:** Acute myocardial infarction, Hospital readmission, Clinical data, Machine learning, Self-adaptive, Stacking-based model learning

## Abstract

**Background:**

Acute myocardial infarction (AMI) is a serious cardiovascular disease, followed by a high readmission rate within 30-days of discharge. Accurate prediction of AMI readmission is a crucial way to identify the high-risk group and optimize the distribution of medical resources.

**Methods:**

In this study, we propose a stacking-based model to predict the risk of 30-day unplanned all-cause hospital readmissions for AMI patients based on clinical data. Firstly, we conducted an under-sampling method of neighborhood cleaning rule (NCR) to alleviate the class imbalance and then utilized a feature selection method of SelectFromModel (SFM) to select effective features. Secondly, we adopted a self-adaptive approach to select base classifiers from eight candidate models according to their performances in datasets. Finally, we constructed a three-layer stacking model in which layer 1 and layer 2 were base-layer and level 3 was meta-layer. The predictions of the base-layer were used to train the meta-layer in order to make the final forecast.

**Results:**

The results show that the proposed model exhibits the highest AUC (0.720), which is higher than that of decision tree (0.681), support vector machine (0.707), random forest (0.701), extra trees (0.709), adaBoost (0.702), bootstrap aggregating (0.704), gradient boosting decision tree (0.710) and extreme gradient enhancement (0.713).

**Conclusion:**

It is evident that our model could effectively predict the risk of 30-day all cause hospital readmissions for AMI patients and provide decision support for the administration.

## Background

Acute myocardial infarction (AMI) is a critical global health issue which causes more than 7 million deaths worldwide per year [1]. According to the evaluation of Healthcare Cost and Utilization Project (HCUP), approximately one in six patients with AMI would have readmission within 30 days of discharge [2]. The high readmission rate poses a tremendous burden on both the patient and the healthcare system. There is an increasing interest in the rate of readmission as an indicator of the quality of hospital care and prognosis of patients [3]. Effective prediction of 30-days all-cause readmission for AMI patients is capable of identifying patients with high risk for readmission, maximizing the potential for successful intervention, and simultaneously optimizing the allocation of scarce medical resources [4, 5].

To date, several methods have been applied to predict the risk of readmission. The most commonly used one is the LACE index, a simple yet effective tool with four attributes including L (Length of stay), A (Acuity of the admission), C (Comorbidity) and E (Emergency department visits) [6]. However, Cotter et al. [7] concluded that the LACE index performed poorly in predicting 30-day readmission with the area under the receiver operating characteristic curve (AUC) of 0.55, while that of the logistic regression (LR) model was 0.57. Regression analysis method is a process of estimating the probability of target variables given some linear combination of the predictors, and has been widely applied to predict the readmission risk [8, 9]. However, it is difficult to solve the nonlinear problem or multicollinearity among risk factors based on detailed clinical data.

In recent years, machine learning (ML) approach has become a promising technique that can be applied to integrate clinical data and improve the predictive ability of the readmission risk [10–12]. Mortazavi et al. [13] used different ML and regression models to predict 30-day all-cause readmission prediction and found that the AUC of random forest (RF) improved by 17.8% compared with LR. However, the application of ML in predicting readmission for AMI patients based on clinical data is limited. Walsh and Hripcsak [14] compared the performances of regularized regression (LASSO) with support vector machine (SVM) in predicting the readmission risk, concluding that both models performed equally. Gupta et al. [15] conducted a comparative analysis of various ML methods, including SVM, naïve bayes (NB), RF and gradient boosting decision tree (GBDT), in predicting AMI readmission based on 204 routinely available clinical variables. Nevertheless, the results showed that ML models did not provide a discriminative improvement compared with the LACE model and other regression models. Therefore, it is necessary to develop more accurate predictive models for predicting AMI readmission.

Given that each ML approach is likely to be outperform others or flawed in different situations, it is natural to think of a way to integrate multiple ML approaches to get better performance. There are three main ensemble learning methods: Bagging, Boosting, Stacking. Bagging [16], introduced by Breiman, trains several base learners by a different bootstrap sample, then combines them and votes for the final result. Boosting [17], introduced by Freund and Schapire, updates the weights of training data after each training iteration, then combines the classification outputs by weighted votes. Although the voting algorithm (Bagging and Boosting) is the most common in classification tasks, it still belongs to a simple combination strategy, which makes it difficult to find complex information from different classifiers. Stacking technique [18], which uses the predictions of multiple base learners as features to train a new meta learner, is a much more powerful ensemble technique and has been successfully applied in predicting the risk of readmission. Radovanović et al. [19] proposed a framework that integrated domain knowledge in form of hierarchies into LR model through stacking method to forecast readmission of six diseases. The results suggested that the proposed framework improved the AUC by an average of 9% compared with LR model. Yu et al. [20] presented a joint ensemble-learning model, using stacking algorithm to integrate the base ML model and boosting algorithm to predict readmission risk. The results showed that compared with the benchmark method LACE model, the proposed stacking model improved by 22.7% in recall, from 0.726 to 0.891. However, the stacking technique is rarely applied in predicting AMI readmission.

In this study, we attempted to adopt stacking technique to predict the 30-day unplanned all-cause hospital readmissions of patients with AMI based on detailed clinical data. The main contributions of this study are summarized as follows:

- A stacking-based model was proposed to predict AMI readmissions, which has not ever been used in studies of AMI readmission prediction.
- The base classifiers could be self-adaptively selected and applied to the base-layer of the stacking model.

## Methods

### Overview of the research framework

**Fig. 1:**
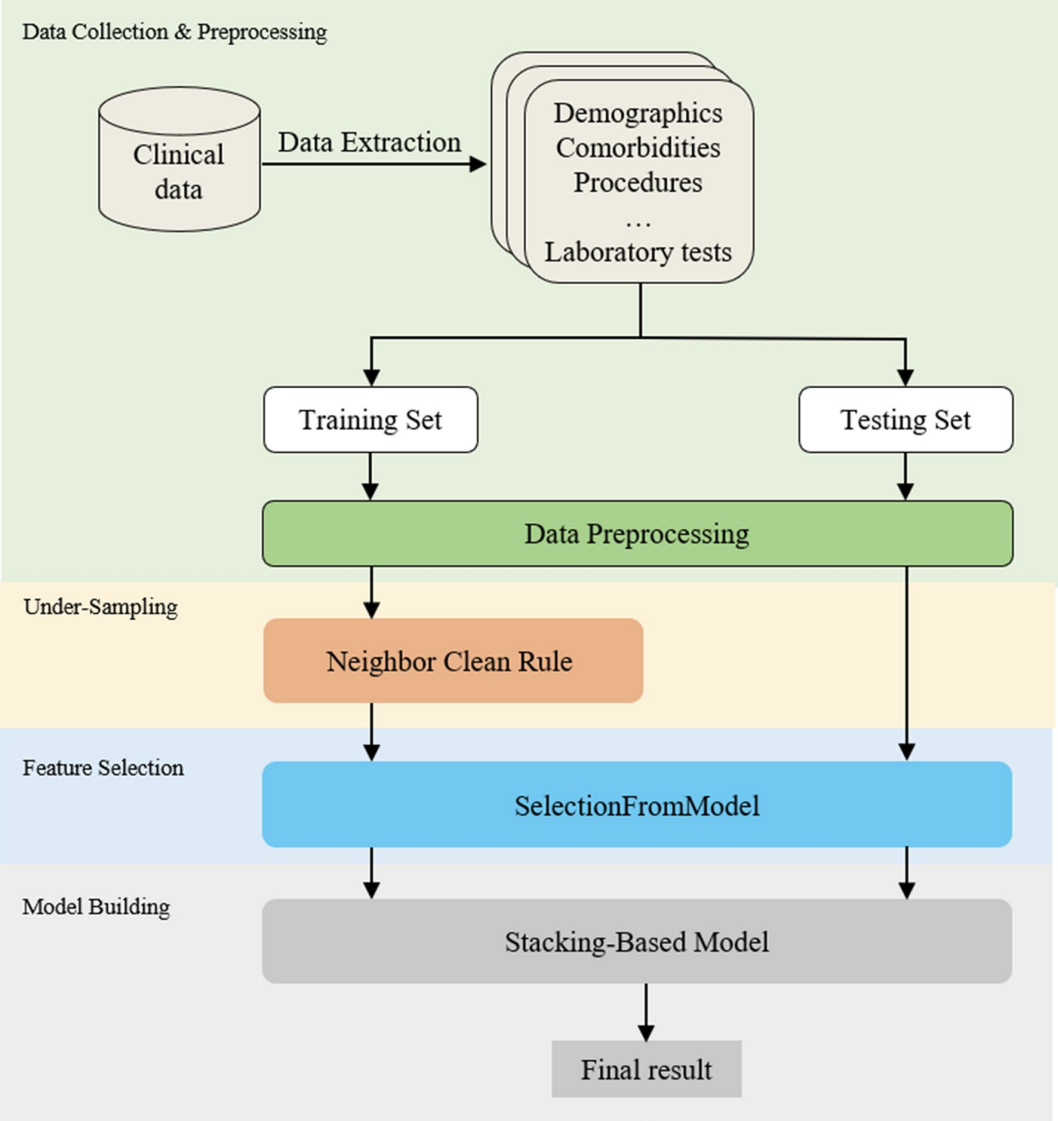
Process flow diagram of the proposed stacking model

The flow diagram of the proposed stacking model is shown in Fig. 1. Firstly, the clinical data were collected and pre-processed. Secondly, an under-sampling method of neighborhood cleaning rule (NCR) was applied to resampling the data. Thirdly, a feature selection method of SelectFromModel (SFM) was utilized to select effective features according the feature importance of each model. Finally, a stacking model based on multiple models was developed for the final prediction.

The details are discussed in the following sub-sections.

### Data collection and preprocessing

#### Data source

Clinical data were derived from West China Hospital, Sichuan University. This study was approved by the Ethics Committee of West China Hospital, Sichuan University (approval no. 2019–165). The Ethics Committee exempted informed consent because of the retrospective nature of this research. Prior to the analysis, patients’ data were anonymized and de-identified.

#### Data extraction

**Fig. 2:**
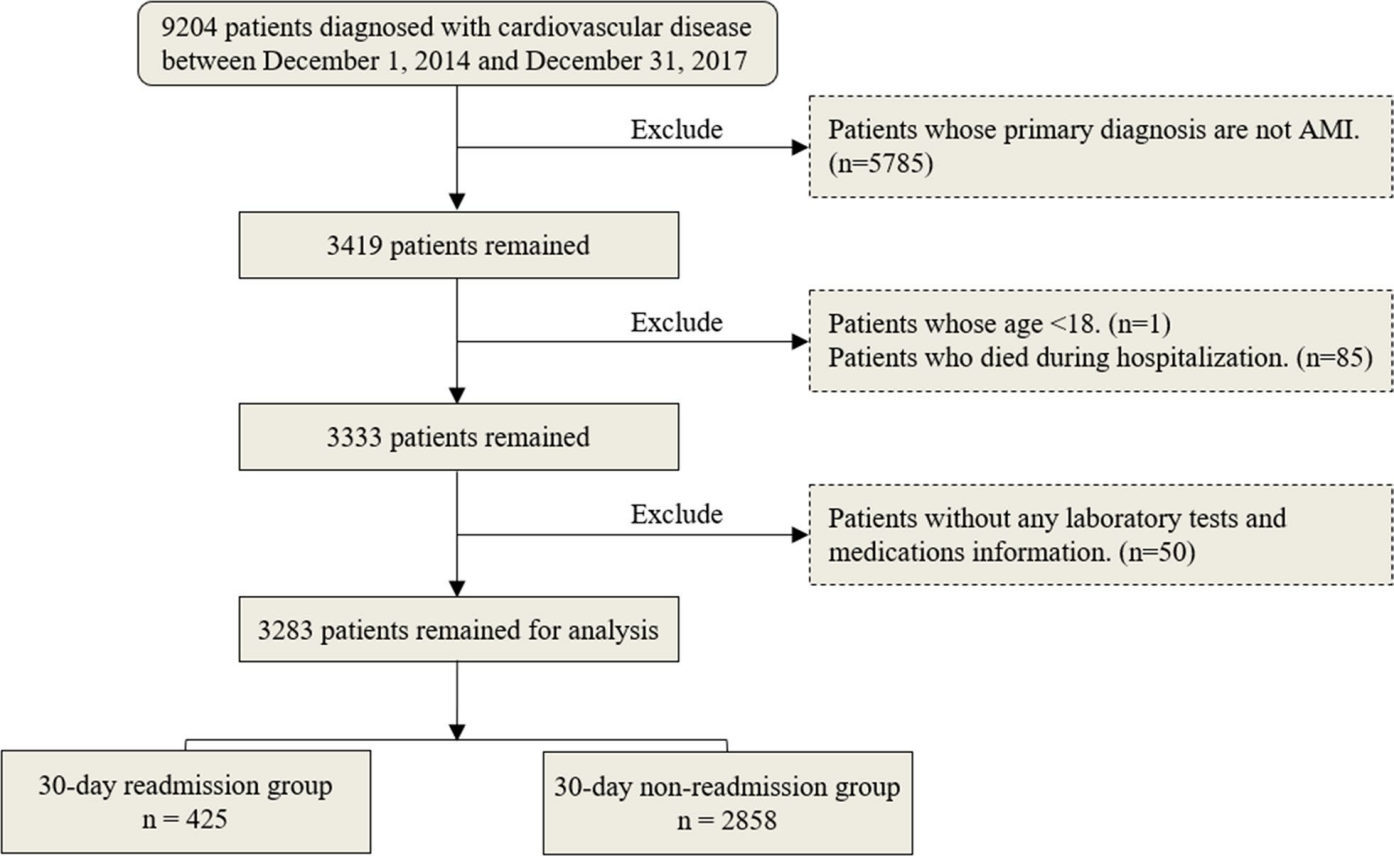
Flow diagram of the selection process

The total samples were from the patients who were diagnosed with cardiovascular disease with discharge dates between December 1, 2014 and December 31, 2017 in West China Hospital, Sichuan University. In this study, we included the patients who were hospitalized for a primary diagnosis of AMI (the 10th revision of the International Statistical Classification of Diseases (ICD-10) Codes: I21.0, I21.1, I21.2, I21.3, I21.4 and I21.9) and excluded the patients who were younger than 18 years, without any laboratory tests and medications information, or died in hospital. In the end, our dataset contains 3283 samples, including 425 readmission samples and 2858 non-readmission samples. Figure 2 shows this study’s patient selection process.

Each record of the data consists of demographics, hospitalization information, medical history, past hospitalization history, comorbidities, physical examinations, procedures, cost information, ultrasonic examinations, laboratory tests and medications. Clinical variables such as some laboratory tests had a low sampling frequency as the result of the lack of necessity in some clinical situations. Here, the variables with more than 20% missing rates were eliminated, because their distributions are difficult to estimate [15, 21, 22]. These discarded variables included some laboratory tests (prothrombin time, fibrinogen, etc.) and physical examinations (height and weight, etc.). For ultrasonic examinations data which were important in cardiovascular disease studies, we categorized those features with a missing rate greater than 30% according to their normal range (e.g., ‘normal’ represents the value within the normal range, ‘abnormalities’ represents the value outside the normal range, ‘unknown’ represents the missing value).

Since one single laboratory test could be performed several times during the medical treatment, the median, min, and max values were calculated to reflect the trend of change to improve the prediction performance. Finally, there were 293 features for analysis. Table 1 shows the various categories of clinical variables, along with the number of variables and some examples. An additional table file shows these clinical variables in more detail (see Additional file 1).

**Table 1:** Description of Clinical variables

Category	Number of variables	Examples
Demographics	8	Sex, Age, Ethnic, Work, Marital status, Address, Physical condition of mother, Physical condition of father
Hospitalization information	5	Length of stay, The month of discharge, Payment Method, Admission condition, Admission pathway
Medical history	5	History of infection, History of trauma, History of surgery, History of allergy, History of blood transfusion
Past hospitalization history	5	Frequency of hospitalizations in the past 1 week, Frequency of hospitalizations in the past 1 month, Frequency of hospitalizations in the past 3 months, Frequency of hospitalizations in the past 6 months, Frequency of hospitalizations in the past 1 year
Comorbidities (ICD-10)	25	e.g., Hypertensive diseases (I10-I15), Diabetes mellitus (E10-E14), Renal failure (N17-N19), Malignant neoplasms (C00-C97), Diseases of liver (K70-K77), The total number of comorbidities
Physical examinations	14	e.g., Heart rate, Respiratory rate, Body temperature, Pulse, Edema, Cardiac Murmurs
Procedures (ICD-9-CM-3)	11	e.g., Procedures on blood vessels (00.6), Angiocardiography using constrast material (88.5), Intravascular imaging of blood vessels (00.2), Puncture of vessel (38.9)
Cost information	17	e.g., Total expenses, Treatment expenses, Western medicine expenses, Bed expenses, Board expenses, Surgery expenses
Ultrasonic examinations	19	e.g., Ejection Fraction, Interventricular septal thickness, Stroke volume
Laboratory tests	168	e.g., Calcium max, Calcium min, Calcium median, Hemoglobin max, Hemoglobin min, Hemoglobin median
Medications	16	e.g., β-receptor blocker, Calcium channel blockers, Angiotensin converting enzyme inhibitors, Angiotensin receptor blocks, Statins, Diuretic

*ICD-10* the 10th revision of the International Statistical Classification of Diseases, *ICD-9-CM-3* International classfication of diseases clinical modification of 9th revision operations and procedures

#### Data preprocessing

Before data preprocessing, the datasets were split into the training set and the testing set by stratified sampling with the ratio of 8: 2 (2626 and 657 samples respectively) in which the proportion of minority samples and majority samples in the training set and testing set was the same.

Data preprocessing included missing data imputation, one-hot encoding and normalization. The details are as follows:

1. Missing data imputation: although variables with more than 20% missing rates have been removed, some variables also have missing values in the dataset. We applied the following imputation strategy. If the missing data belonged to a categorical feature, we replaced it with a new value (e.g., ‘unknown’). If the missing data belonged to a continuous feature, we used the average of the corresponding feature instead.
2. One-hot encoding: considering that the values of the categorical variables were unordered, the categorical variables were encoded as one-hot-encoding vectors. A feature with n categories could be converted into n features, as shown in Eq. (1).

1 $$\left[\begin{array}{c}1\\ {}2\\ {}3\\ {}\begin{array}{l}\dots \\ {}n\end{array}\end{array}\right]=\left[\begin{array}{ccccc}1& 0& 0& \dots & 0\\ {}0& 1& 0& \dots & 0\\ {}0& 0& 1& \dots & 0\\ {}\dots & \dots & \dots & \dots & 0\\ {}0& 0& 0& 0& 1\end{array}\right]$$

3. Normalization: in order to eliminate numerical differences between variables, all variables were normalized to zero mean and unit variance, which can be defined as Eq. (2). *x* is the input feature, mean and *σ* represent the average and standard deviations of the input feature respectively, and *x*^∗^ indicates the output value after normalization.

2 $$\mathrm{x}\ast =\frac{\mathrm{x}\hbox{-} \mathrm{mean}}{\sigma }$$

### Under-sampling

In supervised classification, learning algorithm tends to put more weights over the majority class, thus causing an imbalance problem which may impact the performance of models [23]. Readmission prediction is an essentially imbalanced problem [8]. The level of class imbalance of a dataset is represented by the imbalance ratio (IR), and an IR of 1:5 indicates that for each minority sample there are 5 majority samples. The IR of our dataset was 1:6.72, and it was more imbalanced than the IR of 30-day all-cause readmissions estimated by Jencks et al. [24]. In order to select an appropriate technique to alleviate the class imbalance in our dataset, we made a preliminary experiment using five-fold cross-validation in a training set to compare three class imbalance addressing techniques, including the over-sampling method SMOTE [25], the cost-sensitive method [26] and the under-sampling method NCR. An additional table file shows the comparison results (see Additional file 2), and NCR performed better compared with other class imbalance addressing techniques in most models. Therefore, we applied the under-sampling method NCR [27], which could remove some redundant majority samples from the majority subset. The detailed steps for the NCR treatment are as follows.

**Fig. 3:**
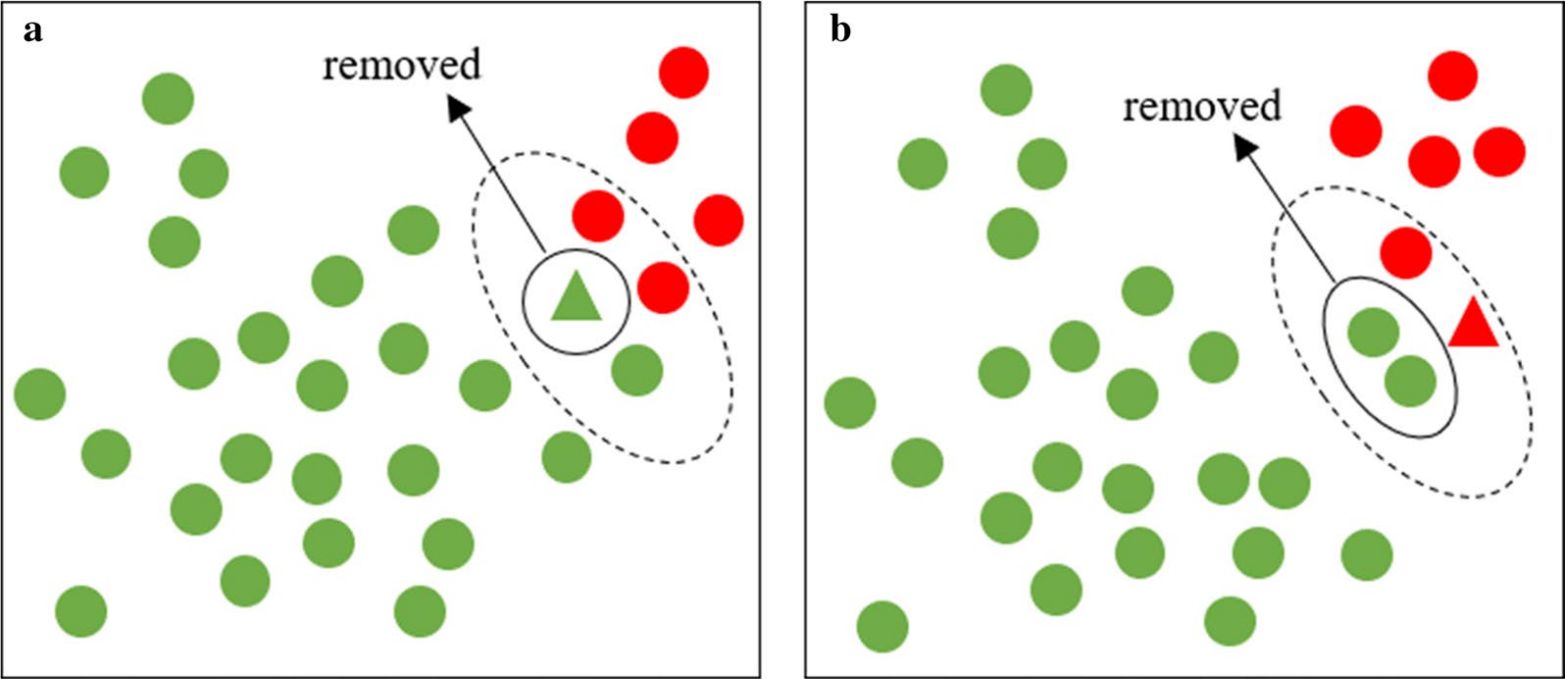
**a** NCR treatment for the sample belongs to the majority subset; **b** NCR treatment for the sample belongs to the minority subset. The green ball represents the majority sample; the red ball represents the minority sample; the green triangle and the red triangle represent the majority and minority samples for analysis, respectively; the samples in the dotted ellipse circle represent the sample to be analyzed and its three closest neighbors

Firstly, find three nearest neighbors for each sample in the training set ***N***. Secondly, as shown in Fig. 3a, if the sample belongs to the majority subset ***N***^**−**^ and at least two of its three nearest neighbors belong to the minority subset ***N***^**+**^, we would remove the sample from the training set. Thirdly, as shown in Fig. 3b, if it belongs to the minority subset ***N***^**+**^, we would remove those of its nearest neighbors that belong to the majority subset ***N***^**−**^ from the training set.

### Feature selection

Feature selection is the process of selecting the optimal feature subset that have important impact on the predicted results [28], which can be efficient to improve model performance and save enormous running time. There are three common feature selection methods: filter, wrapper and embedded [29]. Since the embedded method has better predictive performance than the filter method in general and runs much faster than the wrapper method [30], the embedded method was adopted for our study to select informative variables for the readmission classification. We implemented the embedded method using SFM from scikit-learn package in Python. This method selects features by setting a threshold which is determined by feature importance obtained by training each model on the training set. The features whose feature importance is larger than the threshold would be selected and whose feature importance is smaller than the threshold would be removed. In our study, we traversed all feature subsets according to feature importance of highest to lowest to find the feature subset with the best AUC result. The specific procedures are as follows.

Firstly, the feature importance of all the features are obtained by training the model on the training set. Secondly, set the threshold to the value of the feature importance of each feature, and we could get different feature subsets corresponding to different threshold. Finally, the model performs five-fold cross-validation [31] for each feature subset on the training set to get the average result of AUC for each feature subset, and the feature subset corresponding to the best average result of AUC is the optimal feature subset.

### Model building

Considering that our SFM approach requires the model to have attribute of feature importance, we selected eight broadly representative models as our candidate models, including decision tree (DT), SVM, RF, extra trees (ET), adaBoost (ADB), bootstrap aggregating (Bagging), GBDT, extreme gradient enhancement (XGB) [32–39]. The models’ parameters were optimized with five-fold cross validation, and the values of the parameters for each model are shown in Table 2.

**Table 2:** The parameters of the eight candidate models

Model	Parameters
DT	*max_depth = 10, min_samples_leaf = 20, min_samples_split = 300, random_state = 1*
SVM	*kernel = linear, C = 0.001, tol = 0.0001*
RF	*bootstrap = True, max_depth = 5, n_estimators = 50, random_state = 1*
ET	*bootstrap = False, max_depth = 3, n_estimators = 50, random_state = 1*
GBDT	*learning_rate = 0.05, max_depth = 3, n_estimators = 50, subsample = 0.6, random_state = 1*
ADB	*base_estimator = DecisionTreeClassifier (max_depth = 3), learning_rate = 0.01, n_estimators = 100, random_state = 1*
Bagging	*base_estimator = DecisionTreeClassifier (max_depth = 5), n_estimators = 300, bootstrap = True, max_features = 0.6, max_samples = 0.6, random_state = 1*
XGB	*learning_rate = 0.01, max_depth = 5, n_estimators = 200, subsample = 0.6, colsample_bytree = 0.8, random_state = 1*

*DT* Decision tree, *SVM* Support vector machine, *RF* Random forest, *ET* Extra trees, *GBDT* Gradient boosting decision tree, *ADB* AdaBoost, *Bagging* Bootstrap aggregating, *XGB* Extreme gradient boosting

**Fig. 4:**
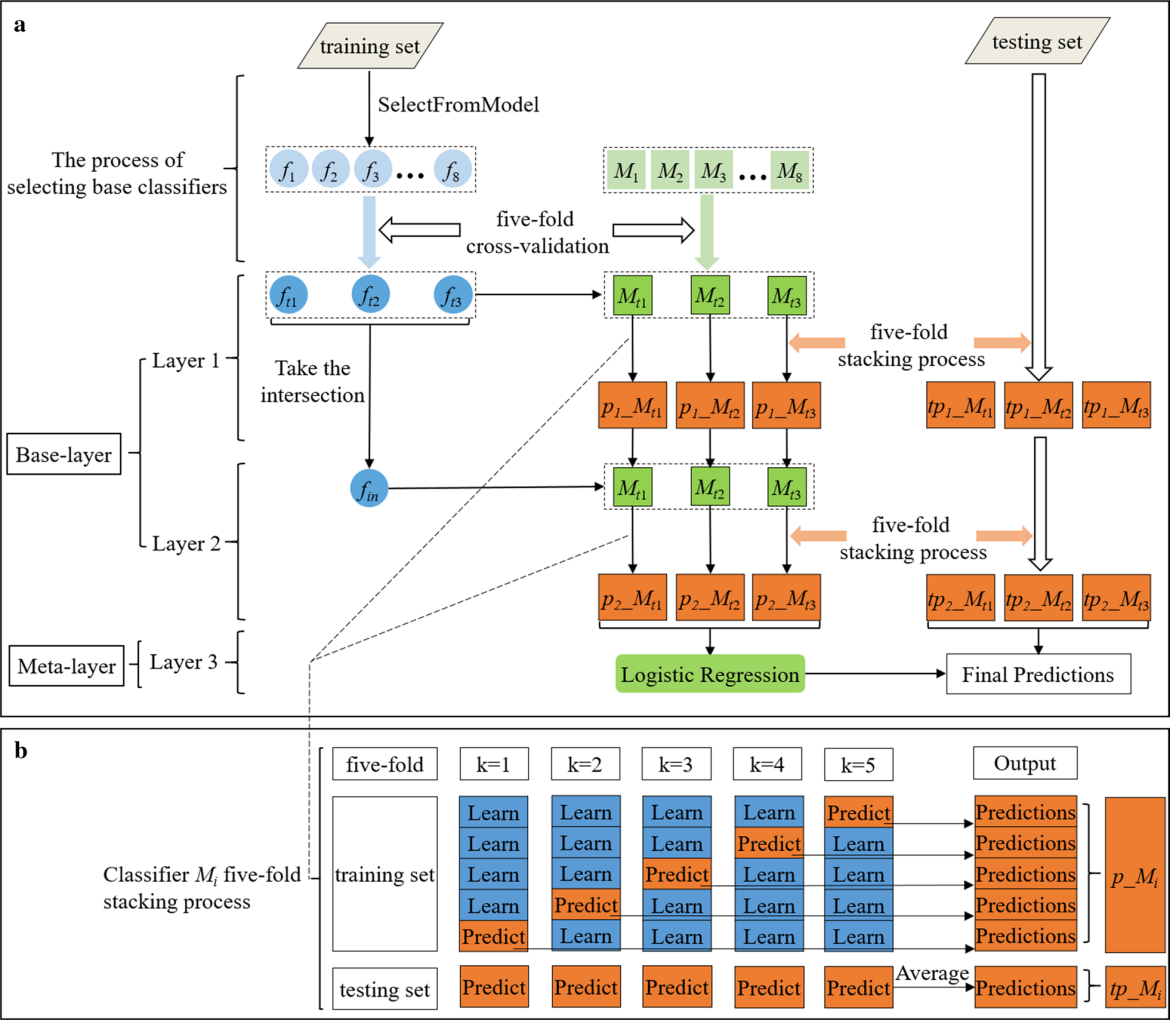
**a** Framework of the stacking-based model; **b** Classifier *M*_*i*_ five-fold stacking process

Firstly, we self-adaptively selected base classifiers for the stacking model. Then, we constructed a three-layer stacking model in which layer 1 and layer 2 were base-layer and level 3 was meta-layer. The base-layer used self-adaptively selected base classifiers to yield predictions by five-fold stacking. Finally, we applied LR for the meta-layer to make the final results based on these predictions. The framework of the stacking-based model is illustrated in Fig. 4a. *M*_1_ to *M*_8_ and *f*_1_ to *f*_8_ represent the eight candidate models and their corresponding feature subsets respectively. *M*_*t*1_ to *M*_*t*3_ and *f*_*t*1_ to *f*_*t*3_ indicate the base classifiers and their corresponding feature subsets respectively. *f*_*in*_ is the intersection of the three feature subsets (*f*_*t*1_ to *f*_*t*3_). *p*_1__*M*_*t*1_ to *p*_1__*M*_*t*3_ indicate the prediction result of the base classifiers in layer 1. *tp*_1__*M*_*t*1_ to *tp*_1__*M*_*t*3_ indicate the average prediction result of the base classifiers in layer 1. *p*_2__*M*_*t*1_ to *p*_2__*M*_*t*3_ represent the prediction result of the base classifiers in layer 2. *tp*_2__*M*_*t*1_ to *tp*_2__*M*_*t*3_ represent the average prediction result of the base classifiers in layer 2. The detailed procedures of the stacking-based model are described as follows.

In the process of adaptively selecting base classifiers, we could get eight candidate models (*M*_1_ to *M*_8_) and their corresponding feature subsets (*f*_1_ to *f*_8_) after feature selection. Then, each of the models applied five-fold cross-validation on their corresponding feature subsets to get the average result of AUC for each model in the training set, and then we selected three models with the best average results of AUC as our base classifiers. The base classifiers (*M*_*t*1_ to *M*_*t*3_) and their corresponding feature subsets (*f*_*t*1_ to *f*_*t*3_) were used to construct the stacking model.

In the first layer, for each selected classifier (*M*_*t*1_ to *M*_*t*3_), with the corresponding feature subsets (*f*_*t*1_ to *f*_*t*3_) as input, the predictions of base classifiers (*p*_1__*M*_*t*1_ to *p*_1__*M*_*t*3_ in training set, *tp*_1__*M*_*t*1_ to *tp*_1__*M*_*t*3_ in testing set) were generated by five-fold stacking. We utilized classifier *M*_*i*_ to illustrate the detailed steps of the five-fold stacking process. As shown in Fig. 4b, we divided the training set into five-fold for cross-validation. In each iteration, four-fold were applied to train classifier, and the remaining one-fold was used for prediction. Meanwhile, in each iteration, the trained classifier predicted testing set. After five iterations, the prediction result for the training set could be obtained (*p*_*M*_*i*_). The average prediction values in testing set were identified as the prediction result of the classifier in testing set (*tp*_*M*_*i*_).

In the second layer, for each base classifier, the input not only included its corresponding generated predictions from the layer 1 (e.g. *p*_1__*M*_*t*1_ generated by *M*_*t*1_ in training set, *tp*_1__*M*_*t*1_ generated by *M*_*t*1_ in testing set), but also additionally added the intersection of the three feature subsets (*f*_*in*_). Then the predictions of base classifiers (*p*_2__*M*_*t*1_ to *p*_2__*M*_*t*3_ in training set, *tp*_2__*M*_*t*1_ to *tp*_2__*M*_*t*3_ in testing set,) were generated by five-fold stacking as mentioned above.

In the third layer, since the features of this layer had been extracted based on complex non-linear transformations, there was no need to choose complex classifiers in the output layer. LR [40] is a good candidate classifier because of its simple structure and the advantage of L2 regularization which can further prevent over-fitting [41]. Therefore, we used LR as the prediction model to train on the training set predictions generated by the layer 2 (*p*_2__*M*_*t*1_ to *p*_2__*M*_*t*3_), and made the final predictions based on the testing set predictions generated by the layer 2 (*tp*_2__*M*_*t*1_ to *tp*_2__*M*_*t*3_).

### Evaluation metrics

According to the systematic review of hospital risk readmission [8], AUC [42] was used as the preferred model evaluation metric in more than 75% of the studies of predicting readmission. In our study, we applied AUC as our main evaluation metric, and took AUC as the performance criterion on which the parameter adjustment and feature selection are based.

In order to further comprehensively compare our proposed model with other models, the evaluation metrics of the confusion matrix were also utilized. Included accuracy, sensitivity and specificity, as shown in (3), (4) and (5), where TP = True Positive, FP = False Positive, TN = True Negative, FN = False Negative.

3 $$\mathrm{Accuracy}=\frac{\mathrm{TP}+\mathrm{TN}}{\mathrm{TP}+\mathrm{TN}+\mathrm{FP}+ FN}$$

4 $$\mathrm{Sensitivity}=\frac{\mathrm{TP}}{\mathrm{TP}+ FN}$$

5 $$\mathrm{Specificity}=\frac{\mathrm{TN}}{\mathrm{TN}+F\mathrm{P}}$$

### Experimental setup

Our project was implemented in Python 3.7.2. Packages of imblearn 0.0 and scikit-learn 0.21 were utilized for under-sampling and feature selection. All the analyses were executed on a computer running the Intel Core i5 3.40 GHz processor, Windows 10 operating system, and 8 GB RAM.

Considering the small sample size of this study and the randomness of the experimental results, we used different random seeds to hierarchically split the dataset for 10 times, and the average result of 10 datasets were applied as the final result. The results were represented in the form of mean ± standard deviation.

## Results

### Results of under-sampling

There were 2626 samples in training set before NCR treatment, of which 2286 were majority. After NCR treatment, there were 1762 majority samples, and 524 redundant majority samples were removed. We used AUC and sensitivity to compare the performances of the eight candidate models between before and after NCR treatment, and the results are shown in Table 3. The average result of AUC for SVM, RF, ET, GBDT, Bagging, XGB were improved after NCR treatment, in which SVM was greatly improved with statistically significant difference (*p*-value < 0.05), while the improvement of other models showed no statistically significant differences. Although the AUC results for most models were not significantly improved after NCR treatment, the sensitivity results for all models were improved and showed statistically significant differences. The results showed the effectiveness of NCR treatment in alleviating the problem of class imbalance.

**Table 3:** The results for the eight candidate models between before and after NCR treatment

Model	AUC		Sensitivity	
	Before	After	Before	After
DT	**0.665 ± 0.03**	0.664 ± 0.02	0.287 ± 0.09	0.385 ± 0.08*
SVM	0.621 ± 0.01	0.662 ± 0.02*	0.161 ± 0.02	0.393 ± 0.03*
RF	0.700 ± 0.02	**0.701 ± 0.02**	0.338 ± 0.04	0.444 ± 0.03*
ET	0.705 ± 0.02	**0.709 ± 0.02**	0.289 ± 0.04	0.453 ± 0.03*
GB	0.698 ± 0.02	**0.702 ± 0.02**	0.338 ± 0.04	0.460 ± 0.04*
ADB	**0.684 ± 0.03**	0.680 ± 0.03	0.351 ± 0.04	0.424 ± 0.04*
Bagging	0.700 ± 0.02	**0.705 ± 0.02**	0.399 ± 0.04	0.498 ± 0.04*
XGB	0.702 ± 0.02	**0.706 ± 0.02**	0.371 ± 0.04	0.468 ± 0.03*

Font bold: the better values; *: there is a statistically significant difference between before and after NCR treatment (*p*-value < 0.05). *DT* Decision tree, *SVM* Support vector machine, *RF* Random forest, *ET* Extra trees, *GBDT* Gradient boosting decision tree, *ADB* AdaBoost, *Bagging* Bootstrap aggregating, *XGB* Extreme gradient boosting

### Results of feature selection

**Fig. 5:**
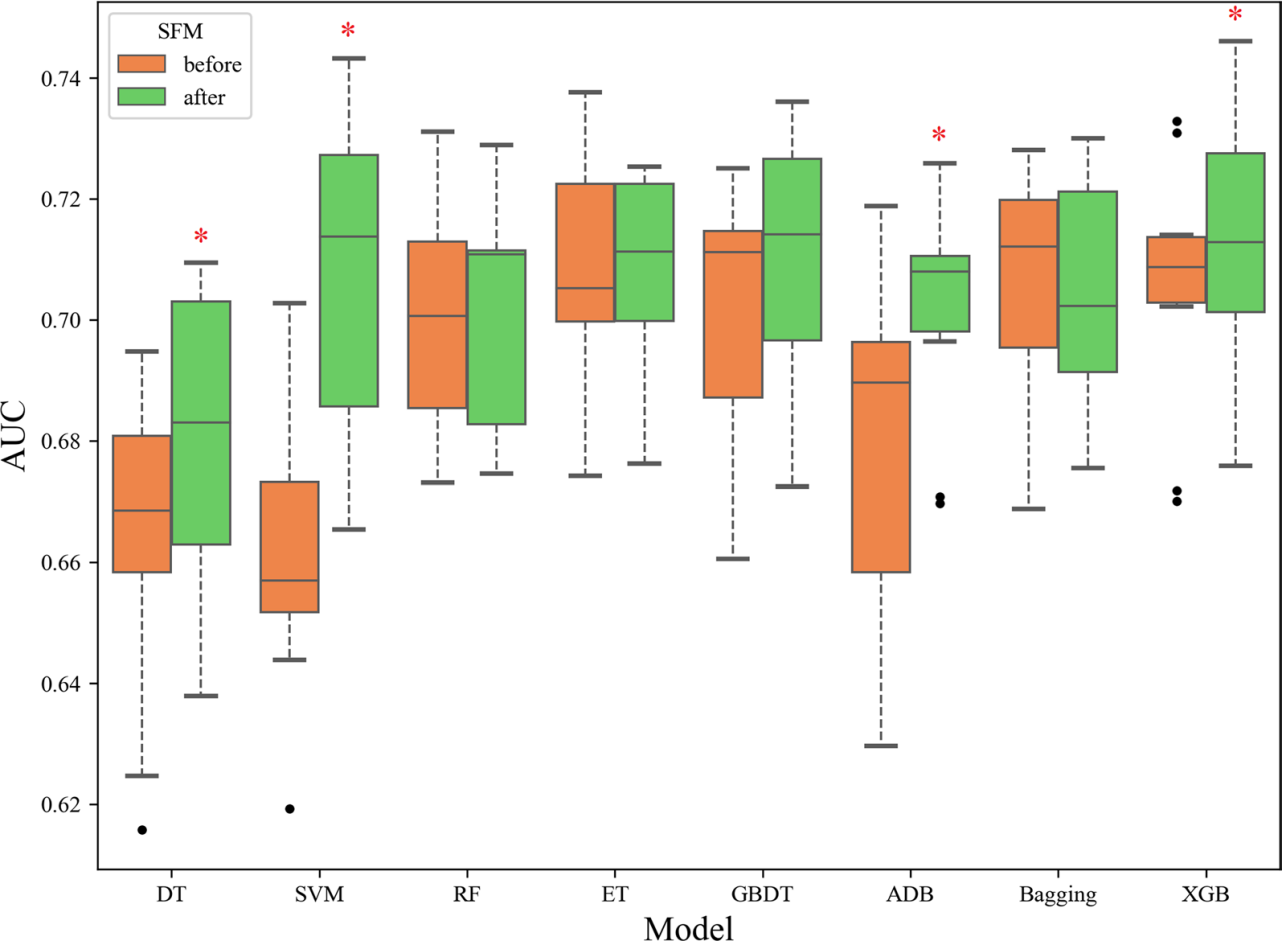
Box plot of the AUC for the eight candidate models between before and after SFM. °: the outliers of box plot, *: there is a statistically significant difference between before and after SFM (*p*-value < 0.05). DT: decision tree; SVM: support vector machine; RF: random forest; ET: extra trees; GBDT: gradient boosting decision tree; ADB: adaBoost; Bagging: bootstrap aggregating; XGB: extreme gradient boosting

There were 392 features in our dataset before feature selection. After SFM, the feature numbers for DT, SVM, RF, ET, GBDT, ADB, Bagging and XGB were 15, 29, 117, 118, 42, 21, 226 and 114, respectively. Each model removed a large number of redundant features, especially for DT, SVM, and ADB, which highly reduced the running time. We used AUC as evaluation metric to compare the performances of the eight candidate models between before and after SFM, and the results are shown in Fig. 5. The results of after SFM showed better performance than that of before SFM in the most of the eight candidate models. Specifically, after SFM, the average result of AUC for DT, SVM, GBDT, ADB, and XGB improved by 2.56, 6.80, 1.14, 3.24 and 0.99%, respectively, and all of them showed statistically significant differences except for GBDT. Although the average result of AUC for Bagging decreased after SFM, it only decreased by 0.001 and with no statistically significant difference. Moreover, after SFM, the outliers of XGB, GB and DT were eliminated, which indicated that SFM could improve the generalization ability of the model. Therefore, for most candidate models, our feature selection method SFM is efficient.

### Results of model comparison

As shown in Table 4, the proposed stacking model achieved higher performance compared with the eight candidate models in all evaluation metrics, in which AUC, accuracy, sensitivity and specificity were 0.720 ± 0.02, 0.772 ± 0.01, 0.515 ± 0.04 and 0.810 ± 0.01, respectively. For AUC, the stacking model improved nearly 1% compared with the best candidate model XGB. For accuracy and sensitivity, compared with the best candidate model in the corresponding evaluation metrics, the stacking model improved by 0.39 and 0.39%, respectively. For specificity, although the stacking model was equal to DT, the standard deviation of the former is less than the latter, which means that the stacking model has better generalization performance than DT.

**Table 4:** Performance comparisons of our stacking model and the eight candidate models

Model	AUC	Accuracy	Sensitivity	Specificity
DT	0.681 ± 0.02	0.768 ± 0.03	0.487 ± 0.06	**0.810 ± 0.04**
SVM	0.707 ± 0.03	0.765 ± 0.01	0.480 ± 0.03	0.808 ± 0.01
RF	0.701 ± 0.02	0.768 ± 0.01	0.502 ± 0.05	0.807 ± 0.01
ET	0.709 ± 0.02	0.760 ± 0.02	0.500 ± 0.03	0.798 ± 0.02
GBDT	0.710 ± 0.02	0.764 ± 0.02	0.501 ± 0.04	0.803 ± 0.02
ADB	0.702 ± 0.02	0.769 ± 0.03	0.502 ± 0.03	0.809 ± 0.03
Bagging	0.704 ± 0.02	0.769 ± 0.01	0.512 ± 0.03	0.808 ± 0.01
XGB	0.713 ± 0.02	0.768 ± 0.02	0.513 ± 0.03	0.806 ± 0.02
**Stacking Model**	**0.720 ± 0.02**	**0.772 ± 0.01**	**0.515 ± 0.04**	**0.810 ± 0.01**

Font bold: the optimal values. *DT* Decision tree, *SVM* Support vector machine, *RF* Random forest, *ET* Extra trees, *GBDT* Gradient boosting decision tree, *ADB* AdaBoost, *Bagging* Bootstrap aggregating, *XGB* Extreme gradient boosting

## Discussion

This study proposes a stacking-based model to predict the risk of 30-day readmission in patients with AMI. The comparison results among the eight candidate models in Table 4 illustrated that the ensemble learning models, including RF, ET, GBDT, ADB, Bagging and XGB outperformed DT and SVM in sensitivity, suggesting that the ensemble learning models perform better to identify minority samples. The specificity of DT was highest among the eight candidate modes, while its AUC and sensitivity were lower, indicating that DT, as a simple machine learning model, functioned in the majority samples of the majority class instead of its prediction effect. The AUC of XGB was best among the eight candidate modes, and it also performed well in other evaluation metrics, which inferred XGB might have outstanding performance in the prediction of readmission [43, 44]. Compared with XGB, the AUC, accuracy, sensitivity and specificity of the proposed stacking model improved by 0.98, 0.52, 0.38 and 0.49%, respectively, suggesting that our model could further improve the overall predictive performance compared with the best individual model. Moreover, compared with the eight candidate models which only performed well in part of evaluation metrics, the stacking model achieved in all of them, indicating that the stacking model could integrate the advantages of different individual models into generate better predictions. The standard deviations of the stacking model in terms of AUC, accuracy, sensitivity and specificity were 0.02, 0.01, 0.04 and 0.01, respectively, among which AUC, accuracy and specificity were the lowest compared with other models, demonstrating that our model had good generalization ability.

However, we also noted that the sensitivity of the stacking model was only 0.515, indicating that the ability of our stacking model to identify the readmitted patients was weak. There are two main reasons for the low sensitivity. On the one hand, the sensitivities of the eight candidate models were low, except for XGB and Bagging which were greater than 0.510, all the other models were around 0.500 or less than 0.500. Stacking technique, which uses the predictions of multiple base learners as features to train a new meta learner, may not achieve a good forecast performance if the base classifiers does not perform well. On the other hand, in our study, the criteria for adjusting the parameters of each candidate model and selecting the base classifiers were based on AUC rather than sensitivity. In addition, we found that some heart failure readmission studies had relatively low sensitivity [10, 13, 45].

In addition to achieve better prediction performances compared with individual ML model, our proposed stacking model has the characteristic of self-adaptively selecting the base classifiers. So far it is an open question that how to obtain an effective combination of base classifiers in stacking methods. Although many studies enumerated all the combinations of classifiers to choose the best combination, it is time-consuming and laborious. In our stacking model, the base classifiers could be automatically selected according to the average results from five-fold cross-validation for the candidate models. The result in Table 4 shows that the base classifiers selection method is effective for constructing the stacking model.

Considering the enormous burden of AMI readmission in the healthcare system [46, 47], accurate prediction of readmission could improve the administration of the hospital and save cost. Therefore, several models have been established to forecast 30-day readmissions in patients with AMI. However, many existing forecasting models use data that is not available until a long time after discharge (e.g., registry or administrative claims) [48], thus limiting their applicability for clinical use. We overcame the limitation by only using data collected during the patient’s hospitalization. Our study included all available clinical data, including demographics, hospitalization information, medical history, past hospitalization history, comorbidities, physical examinations, procedures, cost information, ultrasonic examinations, laboratory tests and medications, and the detailed clinical data from real world were applied to train the predictive model which made our model more convincing. Meanwhile, it is important to identify some important clinical features from these various clinical features. In the second layer of our stacking-based model, for each base classifier, the input not only included its corresponding generated predictions from the layer 1, but also additionally added the intersection of the corresponding feature subsets of the three base classifiers. The features in the intersection of these feature subsets is very important for our study, including age, length of stay, some cost information, and some laboratory tests. Our selected features confirmed some of the risk factors known to be influential in studies of readmission prediction, such as age, length of stay [49–51]. We also found other less considered risk factors like cost information, including total expenses, treatment expenses. But our study was not able to examine these factors because few studies used the detailed cost information.

It is quite challenging to compare the outcomes of our stacking-based model with the results of the related works in this field. The primary cause is that different studies have great differences in terms of the dataset and the processing procedure of the dataset. However, comparisons with previous studies are still considered a valuable approach to increase awareness of AMI readmission. Table 5 shows the comparison results from our study and previous works. None of the three previous works used any method for class imbalance, and IR of them ranged from 1:3.76 to 1:5.12. Our study applied NCR to alleviate the class imbalance based on IR of 1:6.72. The result in Table 3 indicates that the effectiveness of NCR treatment in alleviating the problem of class imbalance, and could be applied to more readmission studies. Feature selection, as a process of selecting the optimal feature subset, plays a significant role in improving the prediction performance of the model. Yu et al. [53] and Gupta et al. [15] lacked feature selection in their studies. The feature selection method used by Krumholz et al. [52] was stepwise logistic regression [54] method, which was frequently utilized in clinical research. However, its use is disputed to some extent because it relies on automatic feature selection that often takes advantage of random chance factors in a given sample. The feature selection method applied in our study was SFM, which has the characteristics of fast running speed. The result shown in Fig. 5 also indicates that SFM is effective on selecting important risk factors. The three previous studies respectively applied regression analysis method such as LR, linear-SVM and GBDT as the predictive models, and their AUCs ranged from 0.630 to 0.660. The AUC of our stacking model reaches 0.720, demonstrating that our model has better prediction performance than other models. Meanwhile, considering that the sample of this study is relatively small compared with other studies, it has a great influence on the prediction effect of the model. Therefore, the comparison results of AUC also indicate that our stacking model has good predictive performance on relatively small datasets.

**Table 5:** Comparison of our study and previous works

Author	Balance method	Feature selection	Samples	Variables	IR	Method	AUC
Krumholz [52]	no	Stepwise Logistic Regression	200,750	103	1:4.29	LR	0.630
Yu [53]	no	no	844	unknown	1:3.76	linear-SVM	0.660
Gupta [15]	no	no	7018	192	1:5.12	GBDT	0.641
Ours	NCR	SFM	3283	293	1:6.72	Stacking	0.720

IR Imbalance ratio, *NCR* Neighborhood clean rule, *SFM* SelectFromModel, *LR* Logistic regression, *linear-SVM*, Linear support vector machine, *GBDT* Gradient boosting decision tree

Our study has some limitations that need to be addressed. First, since the feature selection method of SFM needs the attribute of feature importance, the model without the attribute of feature importance is not included in this study (e.g. artificial neural network, nonlinear kernel SVM), limiting the ability to compare with more different types of models. Second, some long text information were not included in this study (e.g. history of present illness, discharge summary), otherwise we could get the information about the time of the patient’s illness and the changes of some indicators during the patient’s hospitalization, and this information, thus further improving the model accuracy. Third, in our study, we only used data collected from the patient’s hospitalization. Although this may help hospitals to perform post-hospital interventions, it is evident that some specific interventions may be more valid in decreasing readmission if they were properly performed before discharge [48, 55].

## Conclusions

This study proposes a stacking-based model to predict the risk of 30-day unplanned all-cause hospital readmissions of patients with AMI based on clinical data. Compared with general stacking model, the proposed stacking model has the characteristic of self-adaptively selecting the base classifiers. The comparison results of different models showed that our model was superior to the individual model in all evaluation metrics, demonstrating that the stacking model could integrate the advantages of different individual models to achieve better prediction performance. Moreover, detailed clinical data from real world were used to develop the proposed stacking-based model which made our model more convincing.

Effective readmission risk prediction models could provide the administration with valuable insights to identify high-risk patients and target them for early clinical interventions to reduce the probability of readmission. In future studies, the proposed stacking-based model could also be evaluated with more data from multi-health centers.

## Supplementary Information

**Additional file 1.** Detailed clinical variables.

**Additional file 2.** The results of five-fold cross-validation on the training set of the eight candidate models in different class imbalance treatment techniques.

## Data Availability

The data that support the findings of this study are available from West China Hospital, Sichuan University but restrictions apply to the availability of these data, which were used under license for the current study, and so are not publicly available.

## Abbreviations

AMI: Acute myocardial infarction
AUC: Area under the receiver operating characteristic curve;
ADB: AdaBoost
Bagging: Bootstrap aggregating
DT: Decision tree
ET: Extra trees
GBDT: Gradient boosting decision tree
HCUP: Healthcare Cost and Utilization Project
ICD-10: The 10th revision of the International Statistical Classification of Diseases
ICD-9-CM-3: International classfication of diseases clinical modification of 9th revision operations and procedures
IR: Imbalance ratio
LR: Logistic regression
ML: Machine learning
NB: Naïve bayes
NCR: Neighborhood cleaning rule
RF: Random forest
LASSO: Regularized regression
SVM: Support vector machine
SFM: SelectFromModel
XGB: Extreme gradient enhancement
