# Supplementary material for: A stacking-based model for predicting 30-day all-cause hospital readmissions of patients with acute myocardial infarction
Source: BMC Med Inform Decis Mak. 2020 Dec 14;20:335. doi: 10.1186/s12911-020-01358-w (PMC7734833; doi:10.1186/s12911-020-01358-w)
Supplement: Supplementary file 1 — Additional file 1. Detailed clinical variables. [file 12911_2020_1358_MOESM1_ESM.pdf]

Additional file 1 - Detailed clinical variables.

Category	Variable name	Range	Missing (n(%))	Notes
**Demographics**	Sex	Male,Female	0 (0%)	
	Age	18-99	0 (0%)	
	Ethnic	Yes,No	0 (0%)	Han nationality or not
	Work	0,1,2…14	24 (0.73%)	Different job categories
	Marital status	Yes,No	2 (0.06%)	
	Address	0,1,2	224 (6.82%)	This city, inside province, outside province
	Physical condition of mother	0,1,2	0 (0%)	Dead, alive, ill
	Physical condition of father	0,1,2	0 (0%)	Dead, alive, ill
**Hospitalization information**	Length of stay (days)	1-103	0 (0%)	
	The month of discharge	1-12	0 (0%)	
	Payment Method	0,1,2…18	0 (0%)	Different payment method
	Admission condition	0,1,2	363 (11.06%)	Danger, urgency, general
	Admission pathway	0,1,2	0 (0%)	Outpatient, emergency, others
**Medical history**	History of infection	Yes,No	7 (0.21%)	
	History of trauma	Yes,No	3 (0.09%)	
	History of surgery	Yes,No	3 (0.09%)	
	History of allergy	Yes,No	7 (0.21%)	
	History of blood transfusion	Yes,No	14 (0.43%)	
**Past hospitalization history**	Frequency of hospitalizations in the past 1 week	0-3	0 (0%)	

	Frequency of hospitalizations in the past 1 month	0-4	0 (0%)	
	Frequency of hospitalizations in the past 3 months	0-7	0 (0%)	
	Frequency of hospitalizations in the past 6 months	0-8	0 (0%)	
	Frequency of hospitalizations in the past 1 year	0-15	0 (0%)	
**Comorbidities (ICD-10)**	Malignant neoplasms (C00-C97)	0-10	0 (0%)	Number of occurrences
	Aplastic and other anaemias (D60-D64)	0-10	0 (0%)	Number of occurrences
	Disorders of thyroid gland (E00-E07)	0-10	0 (0%)	Number of occurrences
	Diabetes mellitus (E10-E14)	0-10	0 (0%)	Number of occurrences
	Metabolic disorders (E70-E90)	0-10	0 (0%)	Number of occurrences
	Episodic and paroxysmal disorders (G40-G47)	0-10	0 (0%)	Number of occurrences
	Hypertensive diseases (I10-I15)	0-10	0 (0%)	Number of occurrences
	Cerebrovascular diseases (I60-I69)	0-10	0 (0%)	Number of occurrences
	Diseases of arteries, arterioles and capillaries (I70-I79)	0-10	0 (0%)	Number of occurrences
	Influenza and pneumonia (J09-J18)	0-10	0 (0%)	Number of occurrences
	Chronic lower respiratory diseases (J40-J47)	0-10	0 (0%)	Number of occurrences
	Other diseases of pleura (J90-J94)	0-10	0 (0%)	Number of occurrences
	Other diseases of the respiratory system (J95-J99)	0-10	0 (0%)	Number of occurrences
	Diseases of oesophagus, stomach and duodenum (K20-K31)	0-10	0 (0%)	Number of occurrences
	Diseases of liver (K70-K77)	0-10	0 (0%)	Number of occurrences
	Disorders of gallbladder, biliary tract and pancreas (K80-K87)	0-10	0 (0%)	Number of occurrences
	Other diseases of the digestive system (K90-K93)	0-10	0 (0%)	Number of occurrences
	Arthropathies (M00-M25)	0-10	0 (0%)	Number of occurrences
	Dorsopathies (M40-M54)	0-10	0 (0%)	Number of occurrences

	Renal failure (N17-N19)	0-10	0 (0%)	Number of occurrences
	Diseases of male genital organs (N40-N51)	0-10	0 (0%)	Number of occurrences
	Symptoms and signs involving the circulatory and respiratory systems (R00-R09)	0-10	0 (0%)	Number of occurrences
	General symptoms and signs (R50-R69)	0-10	0 (0%)	Number of occurrences
	Abnormal findings on diagnostic imaging and in function studies, without diagnosis (R90-R94)	0-10	0 (0%)	Number of occurrences
	Persons with potential health hazards related to family and personal history and certain conditions influencing health status (Z80-Z99)	0-10	0 (0%)	Number of occurrences
**Procedures (ICD-9-CM-3)**	Adjunct vascular system procedures (00.4)	0-10	0 (0%)	Number of occurrences
	Removal of coronary artery obstruction and insertion of stent(s) (36.0)	0-10	0 (0%)	Number of occurrences
	Procedures on blood vessels (00.6)	0-10	0 (0%)	Number of occurrences
	Diagnostic procedures on heart and pericardium (00.6)	0-10	0 (0%)	Number of occurrences
	Angiocardiography using constrast material (88.5)	0-10	0 (0%)	Number of occurrences
	Implantation of heart and circulatory assist system(s) (37.6)	0-10	0 (0%)	Number of occurrences
	Other continuous invasive mechanical ventilation (96.7)	0-10	0 (0%)	Number of occurrences
	Nonoperative intubation of gastrointestinal and respiratory tracts (96.0)	0-10	0 (0%)	Number of occurrences
	Insertion, revision, replacement, and removal of leads; insertion of temporary pacemaker system; or revision of cardiac device pocket (37.7)	0-10	0 (0%)	Number of occurrences
	Intravascular imaging of blood vessels (00.2)	0-10	0 (0%)	Number of occurrences
	Puncture of vessel (38.9)	0-10	0 (0%)	Number of occurrences
**Physical examinations**	Heart rate (Median (1th, 3th quartile))	78 (68,89)	175 (5.33%)	
	Respiratory rate (Median (1th, 3th quartile))	20 (19,20)	7 (0.21%)	

	Body temperature (Median (1th, 3th quartile))	36.4 (36.2,36.5)	9 (0.27%)	
	Pulse (Median (1th, 3th quartile))	78 (68,89)	2 (0.06%)	
	Edema	Yes,No	1 (0.03%)	
	Subcutaneous hemorrhage	Yes,No	0 (0%)	
	Cardiac Murmurs	Yes,No	0 (0%)	
	Systolic pressure (Median (1th, 3th quartile))	122 (107,138)	8 (0.24%)	
	Diastolic pressure (Median (1th, 3th quartile))	73 (64,83)	8 (0.24%)	
	Heart boundary	0,1…4	0 (0%)	
	First heart sound	0,1…5	0 (0%)	
	Second heart sound	0,1…4	0 (0%)	
	Arrhythmia	Yes,No	0 (0%)	
	Centimeter distance between heart and sternum (Median (1th, 3th quartile))	7.5 (7.5,7,5)	207 (6.31%)	
**Ultrasonic examinations**	Ejection Fraction (%) (Median (1th, 3th quartile))	55 (45,62)	408 (12.43%)	
	Left ventricle (mm) (Median (1th, 3th quartile))	50 (46,54)	414 (12.61%)	
	Left atrium (mm) (Median (1th, 3th quartile))	36 (32,40)	562 (17.12%)	
	End-diastolic volume (ml) (Median (1th, 3th quartile))	106 (90,126)	665 (20.26%)	
	Stroke volume (ml) (Median (1th, 3th quartile))	57 (47,69)	668 (20.35%)	
	End-systolic volume (ml) (Median (1th, 3th quartile))	47 (35,63)	681 (20.74%)	
	Interventricular septal thickness (mm) (Median (1th, 3th quartile))	10 (9,12)	871 (26.53%)	
	Aortic	0,1,2	1976 (60.19%)	Normal, abnormal, not tested
	Aortic orifice velocity	0,1,2	1242 (37.83%)	Normal, abnormal, not tested
	A peak of mitral valve	0,1,2	2111 (64.3%)	Normal, abnormal, not tested
	End-diastolic dimension	0,1,2	2490 (75.85%)	Normal, abnormal, not tested

	End-systolic dimension	0,1,2	2490 (75.85%)	Normal, abnormal, not tested
	Early diastolic velocity of mitral valve	0,1,2	2055 (62.6%)	Normal, abnormal, not tested
	E peak of mitral valve	0,1,2	1705 (51.93%)	Normal, abnormal, not tested
	Left ventricular posterior wall	0,1,2	1376 (41.91%)	Normal, abnormal, not tested
	Main pulmonary artery	0,1,2	2252 (68.6%)	Normal, abnormal, not tested
	Pulmonary valve orifice velocity	0,1,2	2413 (73.5%)	Normal, abnormal, not tested
	Right atrium	0,1,2	1633 (49.74%)	Normal, abnormal, not tested
	Right ventricle	0,1,2	1545 (47.06%)	Normal, abnormal, not tested
**Medicines**	β-receptor blocker	0-2	0 (0%)	Number of occurrences
	Calcium channel blockers	0-3	0 (0%)	Number of occurrences
	Angiotensin converting enzyme inhibitors	0-2	0 (0%)	Number of occurrences
	Angiotensin receptor blocks	0-1	0 (0%)	Number of occurrences
	Statins	0-2	0 (0%)	Number of occurrences
	Diuretic	0-4	0 (0%)	Number of occurrences
	Direct vasodilator	0-6	0 (0%)	Number of occurrences
	Inotropic agents	0-2	0 (0%)	Number of occurrences
	Oral nitroglycerin	0-2	0 (0%)	Number of occurrences
	Other nitroglycerin	0-2	0 (0%)	Number of occurrences
	Aspirin	0-1	0 (0%)	Number of occurrences
	Antiplatelet drugs	0-4	0 (0%)	Number of occurrences
	Anticoagulant drugs	0-4	0 (0%)	Number of occurrences
	Anti-arrhythmia agent	0-3	0 (0%)	Number of occurrences
	Insulin	0-3	0 (0%)	Number of occurrences

	Hypoglycemic drugs	0-3	0 (0%)	Number of occurrences
**Laboratory tests**<br>(Median (1th, 3th quartile))	Absolute value of basophil_max (10^9/L)	0.02 (0.02,0.04)	476 (14.5%)	
	Absolute value of basophil_median (10^9/L)	0.02 (0.01,0.03)	476 (14.5%)	
	Absolute value of basophil_min (10^9/L)	0.02 (0.01,0.03)	476 (14.5%)	
	Absolute value of eosinophil_max (10^9/L)	0.11 (0.05,0.21)	473 (14.41%)	
	Absolute value of eosinophil_median (10^9/L)	0.09 (0.04,0.16)	473 (14.41%)	
	Absolute value of eosinophil_min (10^9/L)	0.06 (0.01,0.13)	473 (14.41%)	
	Absolute value of monocytes_max (10^9/L)	0.63 (0.45,0.87)	463 (14.1%)	
	Absolute value of monocytes_median (10^9/L)	0.57 (0.42,0.75)	463 (14.1%)	
	Absolute value of monocytes_min (10^9/L)	0.5 (0.36,0.67)	463 (14.1%)	
	Absolute value of neutrophils_max (10^9/L)	6.59 (4.66,9.06)	463 (14.1%)	
	Absolute value of neutrophils_median (10^9/L)	5.98 (4.41,7.92)	463 (14.1%)	
	Absolute value of neutrophils_min (10^9/L)	5.22 (3.99,6.9)	463 (14.1%)	
	Alanine aminotransferase_max (IU/L)	40 (25,65)	473 (14.41%)	
	Alanine aminotransferase_median (IU/L)	36 (23,56)	473 (14.41%)	
	Alanine aminotransferase_min (IU/L)	32 (20,49)	473 (14.41%)	
	Albumin_max (g/L)	39.6 (36.8,42.3)	472 (14.38%)	
	Albumin_median (g/L)	38.6 (35.65,41.6)	472 (14.38%)	
	Albumin_min (g/L)	37.9 (34.3,41.2)	472 (14.38%)	
	Alkaline phosphatase_max (IU/L)	82 (67,101.75)	473 (14.41%)	
	Alkaline phosphatase_median (IU/L)	79 (66,96.38)	473 (14.41%)	
	Alkaline phosphatase_min (IU/L)	75 (63,92)	473 (14.41%)	
	Anion gap_max (mmol/L)	19.3 (16.9,21.8)	303 (9.23%)	

	Anion gap_median (mmol/L)	18 (16.1,20.15)	303 (9.23%)	
	Anion gap_min (mmol/L)	16.6 (14.6,18.9)	303 (9.23%)	
	Aspartate aminotransferase_max (IU/L)	66 (33,182)	469 (14.29%)	
	Aspartate aminotransferase_median (IU/L)	54 (29.13,126)	469 (14.29%)	
	Aspartate aminotransferase_min (IU/L)	40 (25,84)	469 (14.29%)	
	AST/ALT_max	1.99 (1.16,3.83)	472 (14.38%)	
	AST/ALT_median	1.63 (1.01,2.93)	472 (14.38%)	
	AST/ALT_min	1.31 (0.85,2.37)	472 (14.38%)	
	Average red blood cell HGB content_max (pg)	30.7 (29.58,31.9)	463 (14.1%)	
	Average red blood cell HGB content_median (pg)	30.5 (29.4,31.7)	463 (14.1%)	
	Average red blood cell HGB content_min (pg)	30.3 (29.2,31.5)	463 (14.1%)	
	Basophil percentage_max (%)	0.3 (0.2,0.5)	476 (14.5%)	
	Basophil percentage_median (%)	0.25 (0.15,0.4)	476 (14.5%)	
	Basophil percentage_min (%)	0.2 (0.1,0.3)	476 (14.5%)	
	Calcium_max (mmol/L)	2.22 (2.13,2.3)	312 (9.5%)	
	Calcium_median (mmol/L)	2.17 (2.09,2.26)	312 (9.5%)	
	Calcium_min (mmol/L)	2.14 (2.04,2.23)	312 (9.5%)	
	Carbon dioxide binding force_max (mmol/L)	24.1 (22,26.4)	302 (9.2%)	
	Carbon dioxide binding force_median (mmol/L)	23.05 (21.1,25)	302 (9.2%)	
	Carbon dioxide binding force_min (mmol/L)	22 (19.7,24.1)	302 (9.2%)	
	Chlorine_max (mmol/L)	104.2 (101.9,106.5)	197 (6%)	
	Chlorine_median (mmol/L)	102.95 (100.4,105.2)	197 (6%)	
	Chlorine_min (mmol/L)	101.7 (98.7,104.3)	197 (6%)	

	Cholesterol_max (mmol/L)	4.06 (3.44,4.79)	496 (15.11%)	
	Cholesterol_median (mmol/L)	3.89 (3.3,4.62)	496 (15.11%)	
	Cholesterol_min (mmol/L)	3.77 (3.14,4.5)	496 (15.11%)	
	Creatine kinase isoenzyme MB mass_max (ng/ml)	15.7 (3.35,112)	150 (4.57%)	
	Creatine kinase isoenzyme MB mass_median (ng/ml)	7.43 (2.61,48.95)	150 (4.57%)	
	Creatine kinase isoenzyme MB mass_min (ng/ml)	3.57 (1.93,14.86)	150 (4.57%)	
	Creatine kinase_max (IU/L)	299 (103.5,1295)	496 (15.11%)	
	Creatine kinase_median (IU/L)	210 (87,802)	496 (15.11%)	
	Creatine kinase_min (IU/L)	141 (70,475.5)	496 (15.11%)	
	Creatinine_max (umol/L)	84 (71,106)	303 (9.23%)	
	Creatinine_median (umol/L)	81 (68.5,99.5)	303 (9.23%)	
	Creatinine_min (umol/L)	77 (65.7,94)	303 (9.23%)	
	Serum cystatin C_max (mg/L)	1.06 (0.92,1.34)	419 (12.76%)	
	Serum cystatin C_median (mg/L)	1.04 (0.9,1.28)	419 (12.76%)	
	Serum cystatin C_min (mg/L)	1.01 (0.88,1.22)	419 (12.76%)	
	Direct bilirubin_max (umol/L)	4.9 (3.6,6.9)	473 (14.41%)	
	Direct bilirubin_median (umol/L)	4.5 (3.35,6.2)	473 (14.41%)	
	Direct bilirubin_min (umol/L)	4.1 (3.1,5.7)	473 (14.41%)	
	Globulin_max (g/L)	26.8 (24.1,30)	472 (14.38%)	
	Globulin_median (g/L)	25.95 (23.4,28.75)	472 (14.38%)	
	Globulin_min (g/L)	25.1 (22.5,27.7)	472 (14.38%)	
	Glucose_max (mmol/L)	7.14 (5.79,10.06)	480 (14.62%)	
	Glucose_median (mmol/L)	6.62 (5.57,8.79)	480 (14.62%)	

	Glucose_min (mmol/L)	6.08 (5.2,7.78)	480 (14.62%)	
	Glutamyl transpeptidase_max (IU/L)	37 (21,74)	473 (14.41%)	
	Glutamyl transpeptidase_median (IU/L)	34.5 (20,66)	473 (14.41%)	
	Glutamyl transpeptidase_min (IU/L)	31 (19,58)	473 (14.41%)	
	Hematocrit_max (L/L)	0.4 (0.37,0.44)	463 (14.1%)	
	Hematocrit_median (L/L)	0.4 (0.36,0.43)	463 (14.1%)	
	Hematocrit_min (L/L)	0.39 (0.34,0.43)	463 (14.1%)	
	Hemoglobin_max (g/L)	133 (120,146)	463 (14.1%)	
	Hemoglobin_median (g/L)	130 (116,143)	463 (14.1%)	
	Hemoglobin_min (g/L)	128 (112,142)	463 (14.1%)	
	High density lipoprotein_max (mmol/L)	1.1 (0.9,1.31)	496 (15.11%)	
	High density lipoprotein_median (mmol/L)	1.04 (0.86,1.26)	496 (15.11%)	
	High density lipoprotein_min (mmol/L)	0.99 (0.8,1.23)	496 (15.11%)	
	Hydroxybutyrate dehydrogenase_max (IU/L)	342 (197,671)	497 (15.14%)	
	Hydroxybutyrate dehydrogenase_median (IU/L)	308 (185.13,577.63)	497 (15.14%)	
	Hydroxybutyrate dehydrogenase_min (IU/L)	269 (171,493)	497 (15.14%)	
	Indirect bilirubin_max (umol/L)	8.3 (5.8,12.1)	473 (14.41%)	
	Indirect bilirubin_median (umol/L)	7.4 (5.1,10.6)	473 (14.41%)	
	Indirect bilirubin_min (umol/L)	6.6 (4.4,9.6)	473 (14.41%)	
	Lactate dehydrogenase_max (IU/L)	380 (231,709)	497 (15.14%)	
	Lactate dehydrogenase_median (IU/L)	343.5 (220,610.75)	497 (15.14%)	
	Lactate dehydrogenase_min (IU/L)	304 (205.25,525.75)	497 (15.14%)	
	Low density lipoprotein_max (mmol/L)	2.3 (1.79,2.9)	496 (15.11%)	

	Low density lipoprotein_median (mmol/L)	2.16 (1.67,2.74)	496 (15.11%)	
	Low density lipoprotein_min (mmol/L)	2.07 (1.56,2.66)	496 (15.11%)	
	Lymphocyte absolute value_max (10^9/L)	1.52 (1.15,1.95)	463 (14.1%)	
	Lymphocyte absolute value_median (10^9/L)	1.39 (1.04,1.82)	463 (14.1%)	
	Lymphocyte absolute value_min (10^9/L)	1.28 (0.89,1.73)	463 (14.1%)	
	Lymphocyte percentage_max (%)	20.1 (14.3,26)	463 (14.1%)	
	Lymphocyte percentage_median (%)	17.6 (12.3,23.7)	463 (14.1%)	
	Lymphocyte percentage_min (%)	15.6 (9.6,22.5)	463 (14.1%)	
	Magnesium_max (mmol/L)	0.88 (0.82,0.94)	312 (9.5%)	
	Magnesium_median (mmol/L)	0.86 (0.81,0.91)	312 (9.5%)	
	Magnesium_min (mmol/L)	0.83 (0.78,0.89)	312 (9.5%)	
	Mean erythrocyte HGB concentration_max (g/L)	332 (324,339)	463 (14.1%)	
	Mean erythrocyte HGB concentration_median (g/L)	329 (322,336)	463 (14.1%)	
	Mean erythrocyte HGB concentration_min (g/L)	327 (319,334)	463 (14.1%)	
	Mean erythrocyte volume_max (fL)	93.2 (90,96.6)	463 (14.1%)	
	Mean erythrocyte volume_median (fL)	92.6 (89.4,96)	463 (14.1%)	
	Mean erythrocyte volume_min (fL)	92 (88.7,95.3)	463 (14.1%)	
	Measurement of serum of lha_max (mmol/L)	0.15 (0.09,0.39)	303 (9.23%)	
	Measurement of serum of lha_median (mmol/L)	0.12 (0.08,0.24)	303 (9.23%)	
	Measurement of serum of lha_min (mmol/L)	0.08 (0.06,0.14)	303 (9.23%)	
	Myoglobin_max (ng/ml)	67.14 (33.1,233.5)	150 (4.57%)	
	Myoglobin_median (ng/ml)	50.53 (29.54,121.47)	150 (4.57%)	
	Myoglobin_min (ng/ml)	37.02 (24.72,66.41)	150 (4.57%)	

	Percentage of eosinophil_max (%)	1.5 (0.6,3)	473 (14.41%)	
	Percentage of eosinophil_median (%)	1.1 (0.4,2.25)	473 (14.41%)	
	Percentage of eosinophil_min (%)	0.7 (0.1,1.8)	473 (14.41%)	
	Percentage of monocytes_max (%)	7.8 (6.2,9.4)	463 (14.1%)	
	Percentage of monocytes_median (%)	7.1 (5.75,8.55)	463 (14.1%)	
	Percentage of monocytes_min (%)	6.4 (4.9,8)	463 (14.1%)	
	Percentage of neutrophils_max (%)	75.3 (67.3,82.9)	463 (14.1%)	
	Percentage of neutrophils_median (%)	72.63 (65.9,78.96)	463 (14.1%)	
	Percentage of neutrophils_min (%)	69.6 (63.4,76.23)	463 (14.1%)	
	Platelet count_max (10^9/L)	179 (137,231)	463 (14.1%)	
	Platelet count_median (10^9/L)	167 (128.38,214)	463 (14.1%)	
	Platelet count_min (10^9/L)	156 (119,202)	463 (14.1%)	
	Potassium_max (mmol/L)	4.26 (3.99,4.59)	197 (6%)	
	Potassium_median (mmol/L)	4.09 (3.87,4.35)	197 (6%)	
	Potassium_min (mmol/L)	3.91 (3.66,4.19)	197 (6%)	
	Proportion of white balls_max	1.55 (1.36,1.76)	472 (14.38%)	
	Proportion of white balls_median	1.48 (1.29,1.7)	472 (14.38%)	
	Proportion of white balls_min	1.44 (1.23,1.64)	472 (14.38%)	
	RBC distribution width CV_max (%)	13.6 (13,14.4)	463 (14.1%)	
	RBC distribution width CV_median (%)	13.4 (12.9,14.25)	463 (14.1%)	
	RBC distribution width CV_min (%)	13.3 (12.8,14.1)	463 (14.1%)	
	RBC distribution width SD_max (fL)	45.8 (43.1,48.9)	463 (14.1%)	
	RBC distribution width SD_median (fL)	45.2 (42.75,48.1)	463 (14.1%)	

	RBC distribution width SD_min (fL)	44.5 (42.2,47.3)	463 (14.1%)	
	Red blood cell count_max (10^12/L)	4.36 (3.95,4.78)	463 (14.1%)	
	Red blood cell count_median (10^12/L)	4.26 (3.81,4.69)	463 (14.1%)	
	Red blood cell count_min (10^12/L)	4.19 (3.69,4.65)	463 (14.1%)	
	Serum inorganic phosphorus_max (mmol/L)	1.06 (0.91,1.23)	311 (9.47%)	
	Serum inorganic phosphorus_median (mmol/L)	0.99 (0.85,1.13)	311 (9.47%)	
	Serum inorganic phosphorus_min (mmol/L)	0.91 (0.76,1.07)	311 (9.47%)	
	Sodium_max (mmol/L)	141 (139.1,143)	197 (6%)	
	Sodium_median (mmol/L)	139.98 (138,141.7)	197 (6%)	
	Sodium_min (mmol/L)	138.9 (136.7,141)	197 (6%)	
	Total protein_max (g/L)	66.3 (62.2,70.1)	472 (14.38%)	
	Total protein_median (g/L)	64.7 (61,68.6)	472 (14.38%)	
	Total protein_min (g/L)	63.4 (59.35,67.7)	472 (14.38%)	
	Urea_max (mmol/L)	6.4 (4.9,9)	419 (12.76%)	
	Urea_median (mmol/L)	6.04 (4.7,8)	419 (12.76%)	
	Urea_min (mmol/L)	5.58 (4.39,7.2)	419 (12.76%)	
	Total bilirubin_max (umol/L)	13.35 (9.7,19.1)	473 (14.41%)	
	Total bilirubin_median (umol/L)	12.1 (8.9,16.99)	473 (14.41%)	
	Total bilirubin_min (umol/L)	11 (7.9,15.4)	473 (14.41%)	
	triglycerides_max (mmol/L)	1.48 (1.1,2.05)	496 (15.11%)	
	triglycerides_median (mmol/L)	1.37 (1.02,1.85)	496 (15.11%)	
	triglycerides_min (mmol/L)	1.25 (0.93,1.72)	496 (15.11%)	
	Troponin -T_max (ng/L)	1828 (408.5,4854)	134 (4.08%)	

	Troponin -T_median (ng/L)	1395 (313.85,3813.75)	134 (4.08%)	
	Troponin -T_min (ng/L)	900.3 (174.1,2736)	134 (4.08%)	
	Urinary sodium_max (pg/ml)	1729 (704.5,4446)	237 (7.22%)	
	Urinary sodium_median (pg/ml)	1460.75 (622.25,3554.5)	237 (7.22%)	
	Urinary sodium_min (pg/ml)	1160 (487,2714)	237 (7.22%)	
	White blood cell count_max (10^9/L)	8.93 (6.94,11.48)	463 (14.1%)	
	White blood cell count_median (10^9/L)	8.28 (6.61,10.31)	463 (14.1%)	
	White blood cell count_min (10^9/L)	7.55 (6.05,9.47)	463 (14.1%)	
**Cost information** (Median (1th, 3th quartile))	Total expenses	47186.6 (30662.9,59242.4)	0 (0%)	
	Treatment expenses	438.38 (207,955.5)	0 (0%)	
	Examination expenses	470 (368,649)	0 (0%)	e.g., Magnetic resonance
	CT (Computed Tomography) expenses	0 (0,0)	0 (0%)	
	Materials expenses	27352.88 (15366.9,42160.1)	0 (0%)	
	Nursing expenses	167 (101,292.1)	0 (0%)	
	Laboratory test expenses	1462.75 (1063,2268.4)	0 (0%)	e.g., Blood routine examination
	Bed expenses	222 (128,411)	0 (0%)	
	Custody expenses	230.05 (118.9,491.6)	0 (0%)	
	Intervention expenses	5630 (4950,5630)	0 (0%)	e.g., The placement of cardiac pacemaker.
	Cooling and heating expenses	12 (6,21)	0 (0%)	
	Surgery expenses	0 (0,0)	0 (0%)	
	Surgical materials expenses	17.2 (0,59.6)	0 (0%)	
	Western medicine expenses	2618.2 (1921,3992.2)	0 (0%)	
	Diagnostic examination expenses	42 (28,70)	0 (0%)	

	Board expenses	133 (48,259.2)	0 (0%)	
	Oxygen expenses	80 (40,209.4)	0 (0%)	

ICD-10: the 10th revision of the International Statistical Classification of Diseases; ICD-9-CM-3: International classfication of diseases clinical modification of 9th revision operations and procedures.
