# Supplementary material for: A stacking-based model for predicting 30-day all-cause hospital readmissions of patients with acute myocardial infarction
Source: BMC Med Inform Decis Mak. 2020 Dec 14;20:335. doi: 10.1186/s12911-020-01358-w (PMC7734833; doi:10.1186/s12911-020-01358-w)
Supplement: Supplementary file 2 — Additional file 2. The results of five-fold cross-validation on the training set of the eight candidate models in different class imbalance treatment techniques. [file 12911_2020_1358_MOESM2_ESM.pdf]

Additional file 2

The results of five-fold cross-validation on the training set of the eight candidate models in different class imbalance treatment techniques.

Model	Cost-sensitive	SMOTE	NCR
DT	0.640±0.05	0.597±0.04	**0.690±0.03**
SVM	**0.694±0.04**	0.655±0.01	0.66±0.02
RF	0.707±0.03	0.702±0.04	**0.709±0.03**
ET	0.711±0.04	0.681±0.03	**0.716±0.03**
GB	0.696±0.03	0.702±0.03	**0.708±0.02**
ADB	0.586±0.03	**0.69±0.03**	0.662±0.04
Bagging	0.701±0.03	0.694±0.03	**0.713±0.03**
XGB	0.692±0.03	0.705±0.04	**0.716±0.02**

font bold: the better values. *DT: decision tree; SVM: support vector machine; RF: random forest; ET: extra trees; GBDT: gradient boosting decision tree; ADB: adaBoost; Bagging: bootstrap aggregating; XGB: extreme gradient boosting*
